# Supplementary material for: TSPO deficiency uncovers a novel role for porphyrins in adipocyte lipid metabolism
Source: J Endocrinol. 2026 Oct 13;271(1):e250187. doi: 10.1530/JOE-25-0187 (PMC13695413; doi:10.1530/JOE-25-0187)
Supplement: Supplementary file 1 [file JOE-25-0187_supplementary_table_1.pdf]

Supplementary Information

Table S1. Primers used for gene expression assays

Gene name		Sequence
Cd36	Forward	5' – GGCTAAATGAGACTGGGAC – 3'
	Reverse	5' – GCAACAACATCACCACTC – 3'
Cpt1a	Forward	5' – CTCAAACCTATTCGTCTTCTG – 3'
	Reverse	5' – TTGGATGGTGTCTGTCCTC – 3'
Hadha	Forward	5' – TGCATTTGCCGCAGCTTTAC – 3'
	Reverse	5' – GTTGGCCCAGATTTCGTTCA – 3'
Fasn	Forward	5' – AGAGATCCCGAGACGCTTCT – 3'
	Reverse	5' – GCCTGGTAGGCATTCTGTAGT – 3'
Srebf1c	Forward	5' – GGAGCCATGGATTGCACATT – 3'
	Reverse	5' – GGCCCGGGAAGTCACTGT – 3'
Acaca	Forward	5' – GACCCTACACTTACTGATGAG – 3'
	Reverse	5' – AAGCAATAAGAACCTGACGAG – 3'
Tbp	Forward	5' – CCTTGTACCCTTCACCAATGAC – 3'
	Reverse	5' – ACAGCCAAGATTCACGGTAGA – 3'
Pparg1	Forward	5' – CTCCAAGAATACCAAAGTGCGA – 3'
	Reverse	5' – GCCTGATGCTTTATCCCCACA – 3'
Pparg2	Forward	5' – TCGCTGATGCACTGCCTATG – 3'
	Reverse	5' – GAGAGGTCCACAGAGCTGATT – 3'
Cebpa	Forward	5' – CAAGAACAGCAACGAGTACCG – 3'
	Reverse	5' – GTCACTGGTCAACTCCAGCAC – 3'
Lpl	Forward	5' – TGGCGTAGCAGGAAGTCTGA – 3'
	Reverse	5' – TGCCTCCATTGGGATAAATGTC – 3'
ap2 (Fabp4)	Forward	5' – AAGGTGAAGAGCATCATAACCCT – 3'
	Reverse	5' – TCACGCCTTTCATAACACATTCC – 3'
Glut4	Forward	5' – GTGACTGGAACACTGGTCCTA – 3'

	Reverse	5’ – CCAGCCACGTTGCATTGTAG – 3’
Hsl	Forward	5’ – GCGCTGGAGGAGTGTTTTT – 3’
	Reverse	5’ – CGCTCTCCAGTTGAACCAAG – 3’
Acrp30	Forward	5’ – TGTTCCTCTTAATCCTGCCCA – 3’
	Reverse	5’ – CCAACCTGCACAAGTTCCCTT – 3’
Lep	Forward	5’ – TTCACACACGCAGTCGGTATC – 3’
	Reverse	5’ – GGCTGGTGAGGACCTGTTG – 3’
